# Supplementary material for: Resolving the full spectrum of human genome variation using Linked-Reads
Source: Genome Res. 2019 Apr;29(4):635–45. doi: 10.1101/gr.234443.118 (PMC6442396; doi:10.1101/gr.234443.118)
Supplement: Supplemental Material [file supp_gr.234443.118_Supplemental_longranger-2.2.zip › longranger-2.2.tar.bz2!/lr2.2-public/lib/stan/cmdstan/stan/doc/api/html/fwd_2functions_2acosh_8hpp_source.html]

Stan: src/stan/agrad/fwd/functions/acosh.hpp Source File


|  |  |
| --- | --- |
|  | Stan  2.5.0  probability, sampling & optimization |


- Main Page
- Related Pages
- Namespaces
- Classes
- Files

- File List
- File Members

All Classes Namespaces Files Functions Variables Typedefs Enumerations Enumerator Friends Macros Pages

- src
- stan
- agrad
- fwd
- functions

acosh.hpp

Go to the documentation of this file.

1 #ifndef STAN\_\_AGRAD\_\_FWD\_\_FUNCTIONS\_\_ACOSH\_HPP

2 #define STAN\_\_AGRAD\_\_FWD\_\_FUNCTIONS\_\_ACOSH\_HPP

3

4 #include <stan/agrad/fwd/fvar.hpp>

5 #include <stan/meta/traits.hpp>

6 #include <stan/math/constants.hpp>

7 #include <stan/math/functions/square.hpp>

8 #include <math.h>

9

10 namespace stan {

11

12  namespace agrad {

13

14  template <typename T>

15  inline

16  fvar<T>

17  acosh(const fvar<T>& x) {

18  using ::acosh;

19  using stan::math::square;

20  using std::sqrt;

21  using stan::math::NOT\_A\_NUMBER;

22  return fvar<T>(acosh(x.val\_),

23  x.d\_ / sqrt(square(x.val\_) - 1));

24  }

25  }

26 }

27 #endif

stan::math::square

T square(const T x)

Return the square of the specified argument.

**Definition:** square.hpp:22

stan::agrad::acosh

fvar< T > acosh(const fvar< T > &x)

**Definition:** acosh.hpp:17

stan::math::NOT\_A\_NUMBER

const double NOT\_A\_NUMBER

(Quiet) not-a-number value.

**Definition:** constants.hpp:53

stan::agrad::fvar

**Definition:** fvar.hpp:13

square.hpp

stan::agrad::acosh

var acosh(const stan::agrad::var &a)

The inverse hyperbolic cosine function for variables (C99).

**Definition:** acosh.hpp:67

stan::agrad::square

fvar< T > square(const fvar< T > &x)

**Definition:** square.hpp:15

traits.hpp

stan::agrad::sqrt

fvar< T > sqrt(const fvar< T > &x)

**Definition:** sqrt.hpp:15

fvar.hpp

stan::agrad::fvar::d\_

T d\_

**Definition:** fvar.hpp:16

constants.hpp

stan::agrad::fvar::val\_

T val\_

**Definition:** fvar.hpp:15


---

[ Stan Home Page ]

*© 2011–2014,
Stan Development Team.*
